# Elucidating the multi-target pharmacological mechanism of Xiaoyandina for the treatment of hepatitis C virus based on bioinformatics and cyberpharmacology studies

**DOI:** 10.1097/MD.0000000000041793

**Published:** 2025-03-14

**Authors:** Dilihuma Dilimulati, Nuerbiye Nueraihemaiti, Sendaer Hailati, Mengyuan Han, Kayisaier Abudurousuli, Nulibiya Maihemuti, Alhar Baishan, Alfira Aikebaier, Maidina Abulizi, Wenting Zhou

**Affiliations:** a Department of Pharmacology, School of Pharmacy, Xinjiang Medical University, Urumqi, Xinjiang, P.R. China; b Xinjiang Key Laboratory of Active Components and Drug Release Technology of Natural Medicines, Urumqi, Xinjiang, China; c Xinjiang Key Laboratory of Biopharmaceuticals and Medical Devices, Urumqi, Xinjiang, China; d Engineering Research Center of Xinjiang and Central Asian Medicine Resources, Ministry of Education, Urumqi, Xinjiang, China.

**Keywords:** hepatitis C virus, molecular docking, molecular dynamic, network pharmacology, Xiaoyandina

## Abstract

An estimated 170 million people worldwide suffer from chronic hepatitis C virus (HCV) infection, which is the main reason for liver transplantation in numerous nations. Traditional Chinese medicine is also frequently employed in medicine to treat HCV. Xiaoyandina is frequently employed in traditional medicine, and which has traditionally been used to cure acute and chronic hepatitis, jaundice, acute and chronic cholecystitis, and acute and chronic cholangitis. The information related to active compounds was retrieved from public databases and through literature review which was later combined with differentially expressed genes obtained through microarray datasets; a compound–target genes–disease network was constructed which uncovered that Kaempferol, Sesamin, and Quercetin decisively contributed to the cell growth and proliferation by affecting STAT1, interleukin-6, and CXCL10 proteins. The molecular docking and molecular dynamics simulation of 50 ns well complemented the binding affinity of the compound and revealed strong stability of predicted compounds at the docked site. In total, compound targets were obtained separately from Traditional Chinese Medicine Systems Pharmacology Database and Analysis Platform. And 27, 10,894, 6, and 20 disease targets were acquired separately based on OMIM, GeneCard, DrugBank, and PharmGkb databases. Then, we constructed the compound–target network and protein–protein interaction network. Three hundred ninety-four differentially expressed genes were observed (231 up-regulated and 163 downregulated genes). Hub genes were screened through survival analysis, including interleukin-6, STAT1, and CXCL10. Finally, molecular docking and molecular dynamics analysis results showed more stable binding between 3 hub genes and the 2 most active compounds Kaempferol and Quercetin. Our research suggests a novel scientific approach for evaluating the multi-component, multi-target impact of XYDN’s active compounds. The present investigation suggested Quercetin, Kaempferol, and Sesamin as possible HCV treatments by combining bioinformatics techniques and network pharmacology. But the findings were not validated in actual patients, so further investigation is needed to confirm the potential use of XYDN towards HCV.

## 1. Introduction

An estimated 170 million individuals worldwide suffer from chronic hepatitis C virus (HCV) infection, which is a leading cause of liver transplantation across various countries.^[1,2]^ HCV infection often leads to severe complications, including hepatocellular carcinoma, fibrosis, cirrhosis, and liver failure, and is a major contributor to chronic liver disease.^[3]^ HCV is a positive-sense, single-stranded RNA virus. The polyprotein is cleaved into 3 structural proteins (E2, core, and E1) and 7 nonstructural proteins (NS2, p7, NS4A, NS3, NS5B, NS4B, and NS5A) through the action of both viral and host proteases.^[4]^ The 21 kDa HCV core protein, the first viral protein expressed during infection, plays a critical role in particle assembly and various stages of the viral life cycle.^[5]^ Current treatments for HCV include NS5A inhibitors, NS3/NS4A protease inhibitors, cyclophilin inhibitors, and nucleoside/nonnucleoside NS5B polymerase inhibitors. While these therapies aim to achieve sustained virological response rates with minimal side effects,^[6]^ their practical implementation remains challenging, particularly in low- and middle-income countries where more than 80% of individuals with HCV reside. The high cost of these therapies often restricts their availability and acceptance.^[7]^

The limitations of existing HCV treatments: including adverse effects, drug interactions, low patient compliance, high costs, and suboptimal efficacy, highlight the urgent need for complementary and alternative therapies that are safer and more effective.^[8]^

Traditional Chinese medicine (TCM) offers a promising complementary approach to HCV management due to its established safety and effectiveness.^[9]^ Both TCM decoctions and proprietary formulations have shown therapeutic potential against HCV.^[10]^ According to TCM principles, HCV-related symptoms correspond to conditions such as “epidemic toxin,” “hypochondriac pain,” and “jaundice.” Xue Boyu categorized HCV into 4 syndromes: liver stagnation and spleen deficiency with blood stasis, heat toxin with blood stasis, qi deficiency with yin damage, and liver and kidney yin deficiency.^[11]^ The treatment strategies involve clearing heat and toxins, nourishing the liver and spleen, and strengthening the body’s defenses while eliminating pathogenic factors. Wang Lingtai proposed a dual strategy combining disease identification and syndrome differentiation. He noted that early-stage chronic HCV is often characterized by excess HCV, the middle stage by liver stagnation and spleen deficiency, and the late stage by kidney deficiency with blood stasis.^[12]^

Xiaoyandina (XYDN), a traditional ethnomedicine, incorporates a blend of natural ingredients known for their therapeutic properties. *Cichorium intybus* (chicory) is widely used for liver-related conditions; *Anchusa officinalis* (bugloss) is recognized for its heat-moistening properties and its ability to regulate abnormal black bile; *Rheum officinale* (rhubarb) is valued for its purgative, anti-inflammatory, and detoxifying effects; *Cuscuta chinensis* (dodder) is known for its antiaging, immunomodulatory, hepatoprotective, hypoglycemic, and endocrine-regulating actions; and *Rosa rugosa* (rose), a traditional qi-regulating herb, is used to alleviate depression, harmonize blood, relieve pain, and more.^[13]^

Preliminary clinical and experimental studies suggest that XYDN is effective in treating various forms of hepatitis, cholecystitis, and urinary tract infections, as well as in increasing antiviral efficacy. This study marks the first attempt to apply bioinformatics to analyze XYDN by constructing a comprehensive “drug–compound–disease–pathway” network. The approach aims to predict potential targets and signaling pathways, assess the binding capacity and stability of active components with these targets, and explore the mechanisms through which XYDN exerts its effects on HCV treatment. The findings are intended to inform clinical strategies for HCV treatment, support the development of novel drugs, and clarify how XYDN utilizes multi-target, multipathway mechanisms to address HCV.

## 2. Materials and methods

### 2.1. Screening for XYDN and hepatitis targets

We gathered and anticipated targets of XYDN utilizing the PubChem Database and Traditional Chinese Medicine Systems Pharmacology Database and Analysis Platform (TCMSP Platform). TCMs are frequently taken orally when used in clinical treatment. ADME-related models including drug-likeness^[14]^ and oral bioavailability^[15]^ primarily impact drug absorption via GIT. Following criteria like oral bioavailability ≥ 30% and drug-likeness ≥ 0.18, the bioactive components were further characterized, and the associated targets for each component were identified The next step was to search for disease targets utilizing the term “Hepatitis C” in the following databases: OMIM Database (An Online Catalog of Human Genes and Genetic Disorders), GeneCards Database (The Human Gene Database), and DrugBank Database under the standards of score ≥ 20.

### 2.2. Network construction

Cytoscape 3.9.1 was used to build the compound–target network in order to import the drug’s active compounds and targets. We employed STRING to obtain the protein interaction data. Next, using Cytoscape 3.9.1, the protein–protein interaction (PPI) network was created.

### 2.3. Differentially expressed genes (DEGs) screening

We acquired information on HCV clinical samples from the GEO database (series: GSE40184, GPL96). Prior to starting therapy at Indiana University School of Medicine, clinical samples were taken from ten individuals infected with genotype 1 hepatitis C. Samples of control blood were drawn from participants who were in good health. The differential analysis was carried out with R Studio, and the cutoff to determine DEGs was set at |log2 (Fold Change)|>0.5 and *P*-value < .05.

### 2.4. Functional enrichment analysis

The 3 components of gene ontology analysis, cellular component, biological process, and molecular function, were utilized to describe the functions of the genes.^[16]^ The Kyoto Encyclopedia of Genes and Gnomes enrichment analysis was employed to determine the hub genes’ bioinformation and system function, which led to the enrichment of biological signaling pathways.^[17]^ We performed both of the aforementioned enrichment analyses by gene cluster using the R package Bioconductor ClusterProfiler after entering the analysis results into R.^[18]^

### 2.5. Molecular docking and ADMET profiling

Subsequently, molecular docking was employed to ascertain whether hub gene associations existed for the core pharmacological compounds identified by network pharmacology. Docking was carried out in the manner mentioned below:

1. We obtained 3 hub genes (interleukin-6 [IL-6], CXCL10, and STAT1) based on the outcomes of the prior screening from Network Pharmacology and Bioinformatics analysis. Using the Protein Database (PDB),^[19]^ the target proteins’ 3D structures were discovered and downloaded in PDB format. Following that, the protein was set up in AutoDock 4.2.6 to add hydrogen and remove water, and the structure was saved in PDBQT format.^[20]^
2. The 3 most active molecules were identified in the screening results above (Kaempferol, Sesamin, and Quercetin). We were able to get the compounds’ protein structures by using the PubChem and TCMSP databases.^[21]^ The protein was altered in AutoDock 4.2.6 to add hydrogen and remove water, following the same procedure as in the previous stage. The ligand files were then saved in PDBQT format.
3. We imported the 2 structural files mentioned above into AutoDock 4.2.6 in order to carry out molecular docking. The PDBQT format was used to compute the minimum binding energy. The OpenBabel program was then used to convert the PDBQT format to the PDB format.^[22]^ Lastly, the molecular docking maps were visualized using PyMOL2.5 software.

Then, SMILES structural notation of 3 active ingredients screened from the pharmacokinetics server pkCSM (http://biosig.unimelb.edu.au/pkcsm/) which is used to predict the drugs’s preclinical absorption, distribution, metabolism, excretion, and toxicity (ADMET) study.

### 2.6. Molecular dynamics (MD) simulation

Using Gromacs 2020, molecular dynamics simulations of the protein complexes were carried out with a total period of 50 ns. The molecular force field used for the kinetic simulations was Amber FF14SB and the system was solubilized using the TIP3 water model. The topology of the proteins was established by Gromacs based on the force field. In order to ensure system charge neutrality, sodium and chloride ions were added to each set of kinetic systems that were put in a cubic box filled with water molecules. Energy minimization for the whole system is achieved using the steepest descent method.^[23]^ Periodic boundary conditions were utilized to model continuous systems, while particle mesh Ewald and PME algorithmic techniques were employed to sustain long-range electrostatic interactions. Each set of systems was subjected to 100 ps of NVT and NPT systematic equilibrium with a pressure of 1.0 atm and a temperature of 300 K. After the equilibrium was completed, finished simulations with a total duration of 50 ns were carried out with a time step of 2 fs, with the simulation trajectory being stored every 100 ps. The simulation results were analyzed by parameters such as RMSD, ASAS, and RMSF.^[24]^

### 2.7. Statistical analysis

Through R language (version 4.0), statistical analyses were carried out and *P* < .05 was deemed significant statistically.

## 3. Results

### 3.1. Introduction to the present study

**Figure 1.**
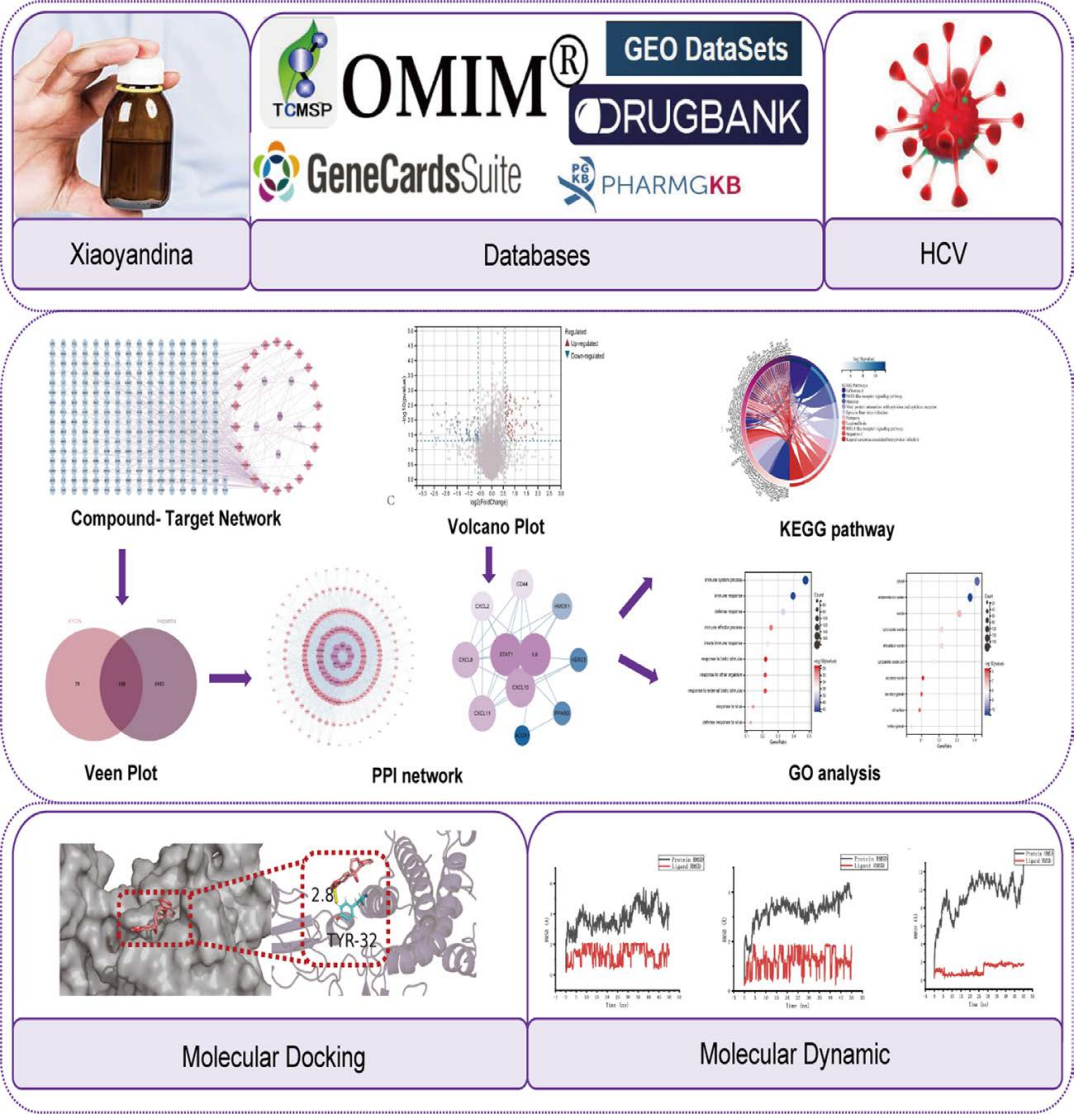
The flow chart of this study.

To investigate HCV treatment, the current study aimed to identify the hub genes targeted by the medication XYDN and examine the associated molecular pathways. Figure 1 presents the flowchart of the investigation. Using databases such as GeneCards (The Human Gene Database) and TCMSP, we first identified the targets of HCV and XYDN. From the GEO database, we analyzed gene expression profiles of healthy and HCV-affected individuals, identifying 394 DEGs with the R limma package. Cross-tabulation analysis revealed 12 overlapping genes, which were subsequently narrowed down to 3 hub genes through the PPI network. Finally, molecular docking using AutoDock software was employed to explore the interactions between the hub genes and the active compounds in greater depth.

### 3.2. XYDN and hepatitis targets collection

Through the TCMSP database, we identified compounds from Cichorii Radix, Cuscutae Semen, Radix Rhei Et Rhizome, Rose Rugosae Flos, and Plantaginis Herba (Table 1). Additionally, by searching the OMIM, GeneCard, DrugBank, and PharmGkb databases, we obtained 27, 10,894, 6, and 20 disease targets, respectively.

**Table 1:** Active compound, their oral bioavailiability (OB), and drug-likeness (DL).

No.	Plant source	Active compounds	Oral bioavailability (OB ≥ 30%)	Drug-likeness (DL ≥ 0.18)
1	Cichorii Radix	Poriferast-5-en-3beta-ol	36.91	0.75
		Beta-sitosterol	36.91	0.75
		Delphinidin	40.63	0.28
		Eseramine	45.89	0.31
		Luteolin	36.16	0.25
		2S,2’S-Aurantiamide acetate	39.18	0.54
		Lactucopicrin	95.31	0.71
		(2R)-3-[3-(5-allyl-2-hydroxyphenyl)-4-hydroxyphenyl]propane-1,2-diol	32.21	0.2
		Gitoxigenin	43.93	0.75
		Cichorioside B	32.05	0.8
		Cyanidin 3-glucoside_qt	58.99	0.24
		ψ-Taraxasterol	39.75	0.76
2	Cuscutae Semen	Sesamin	56.55	0.83
		NSC63551	39.25	0.76
		Isorhamnetin	49.6	0.31
		Beta-sitosterol	36.91	0.75
		Kaempferol	41.88	0.24
		Campest-5-en-3beta-ol	37.58	0.71
		Isofucosterol	43.78	0.76
		Matrine	63.77	0.25
		Sophranol	55.42	0.28
		CLR	37.87	0.68
		Quercetin	46.43	0.28
3	Radix Rhei Et Rhizome	EUPATIN	50.8	0.41
		Mutatochrome	48.64	0.61
		Physciondiglucoside	41.65	0.63
		Procyanidin B-5,3’-O-gallate	31.99	0.32
		Rhein	47.07	0.28
		Sennoside E_qt	50.69	0.61
		Torachrysone-8-O-beta-D-(6’-oxayl)-glucoside	43.02	0.74
		Toralactone	46.46	0.24
		Emodin-1-O-beta-D-glucopyranoside	44.81	0.8
		Sennoside D_qt	61.06	0.61
		Daucosterol_qt	35.89	0.7
		Palmidin A	32.45	0.65
		Beta-sitosterol	36.91	0.75
		Aloe-emodin	83.38	0.24
		Gallic acid-3-O-(6’-O-galloyl)-glucoside	30.25	0.67
		(-)-Catechin	49.68	0.24
4	Rose Rugosae Flos	LYC	32.57	0.51
		Rubixanthin	47.26	0.53
		Rugosin D_qt	57.29	0.67
		Beta-sitosterol	36.91	0.75
		Sitosterol	36.91	0.75
		Cyanin	47.42	0.76
		Vitamin-e	32.29	0.7
		Demethoxycapillarisin	52.33	0.25
		Beta-carotene	37.18	0.58
		Quercetin	46.43	0.28
5	Plantaginis Herba	Dinatin	30.97	0.27
		Baicalein	33.52	0.21
		Baicalin	40.12	0.75
		Sitosterol	36.91	0.75
		6-OH-Luteolin	46.93	0.28
		Stigmasterol	43.83	0.76
		Luteolin	36.16	0.25
		Melampyroside	57.5	0.8
		Stigmasteryl palmitate	38.09	0.4
		β-Sitosteryl palmitate	30.91	0.4

### 3.3. Compound–target network and PPI network construction

**Figure 2.**
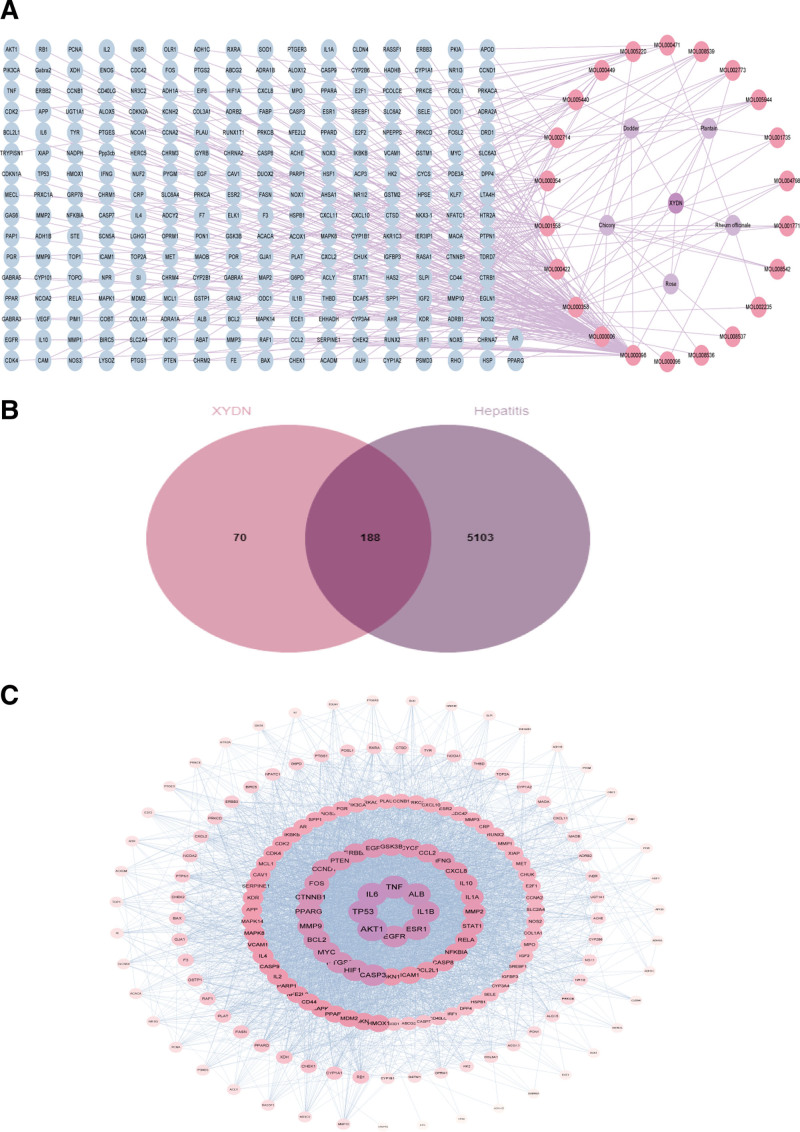
(A) Compound–target network, the size of the compounds was sorted by degree value, and the color of the targets was sorted by degree value. (B) Venn diagram, screening for overlapping genes. (C) PPI network, the protein size according to the degree value. PPI = protein–protein interaction.

Using Cytoscape 3.9.1, a compound–target network was constructed based on 156 targets, 22 active compounds, and 5 components derived from traditional Chinese medicine (Fig. 2A). This network comprises 156 compound targets, 22 compounds, and 5 medicinal components. The compounds were ranked according to their degree value, which reflects their involvement in biological functions and their relative importance. A higher degree value indicates broader biological significance.^[25]^ Concurrently, a Venn diagram analysis identified 188 overlapping drug–disease genes (Fig. 2B). PPI data were retrieved using the STRING database and subsequently imported into Cytoscape 3.9.1 to generate a PPI network diagram (Fig. 2C). Protein size within the diagram was adjusted according to the degree value to reflect their connectivity and significance.

### 3.4. Acquisition of DEGs

**Figure 3.**
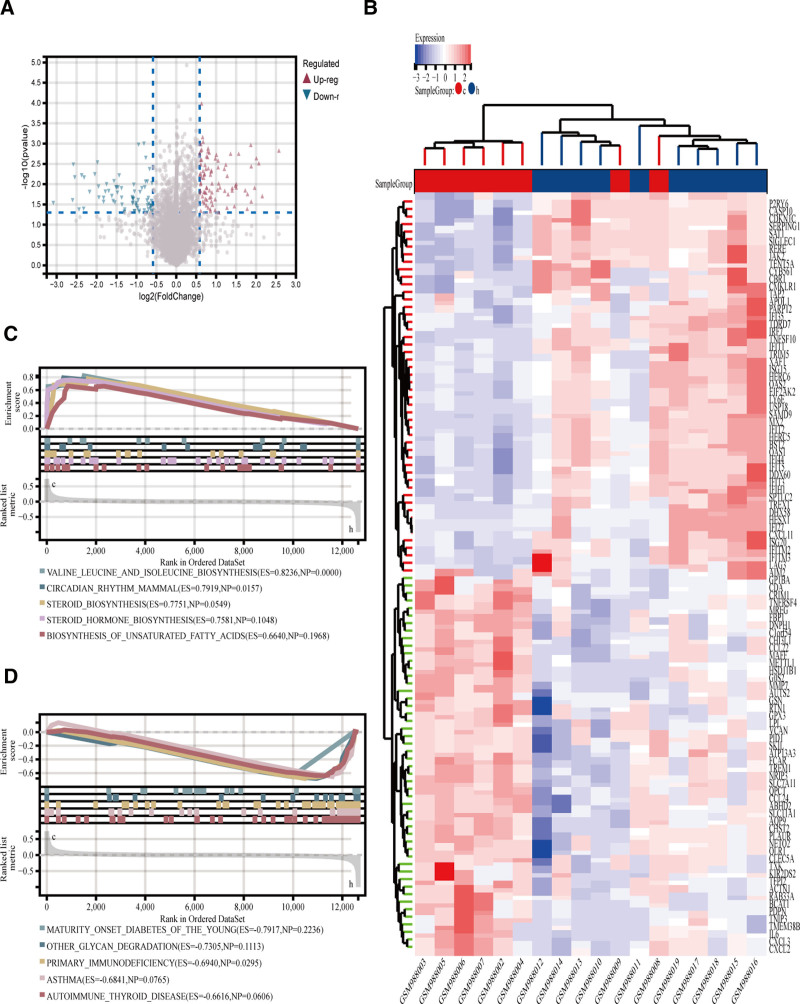
(A) Volcano plot of DEGs, the red color represented up-regulated genes and the blue represented up-regulated genes. (B) Heatmap. (C and D) GSEA plots. DEG = differentially expressed gene.

RNA and hybridization data from blood samples of HCV patients were extracted from the GEO database, and microRNA DEGs were analyzed using R Studio. A total of 394 DEGs were identified, comprising 231 up-regulated and 163 downregulated genes. To illustrate the DEG distribution, a heatmap (Fig. 3B) and a volcano plot (Fig. 3A) were generated. GSEA revealed that these DEGs are associated with pathways such as the RIG-I-like receptor signaling pathway, NOD-like receptor signaling pathway, cell cycle regulation, and primary immunodeficiency (Fig. 3C and D).

**Figure 4.**
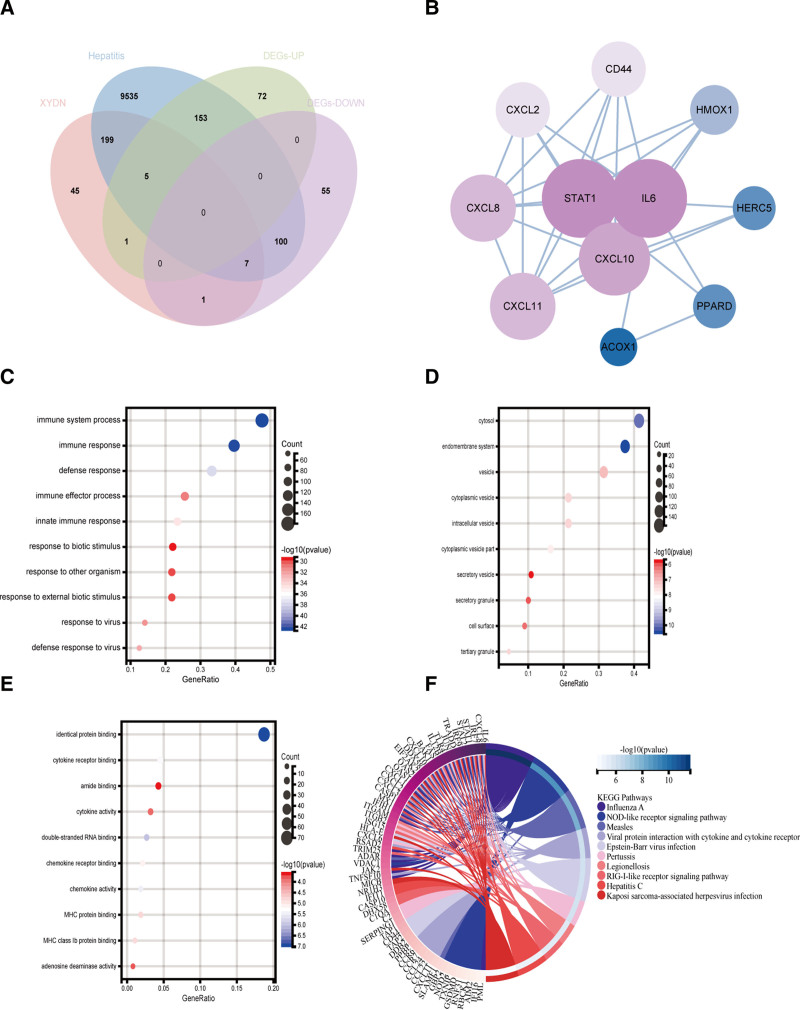
(A) Venn diagram, screening for overlapping genes of XYDN, HCV, and DEGs. (B) PPI network of hub genes. (C) GO analysis – BP terms. (D) GO analysis – CC terms. (E) GO analysis – MF terms. (F) KEGG pathway plot. BP = biological process, CC = cellular component, DEGS = differentially expressed genes, HCV = hepatitis C virus, MF = molecular function, PPI = protein–protein interaction, XYDN = Xiaoyandina.

Subsequently, core genes for XYDN treatment of HCV were identified through overlapping analysis, resulting in 11 key genes (Fig. 4A). Three coding genes were further pinpointed using the PPI network (Fig. 4B), with details of these nodes provided in Table 2.

**Table 2:** Interaction network data of overlap hub genes.

No.	Name	Degree	Closeness	Betweenness
1	STAT1	9.0	9.5	14.56667
2	IL-6	9.0	9.5	22.4
3	CXCL10	8.0	9.0	6.06667
4	CXCL11	7.0	8.5	3.56667
5	CXCL8	7.0	8.5	1.9
6	CXCL2	5.0	7.5	0.0
7	CD44	5.0	7.5	0.0
8	HMOX1	4.0	7.0	0.0
9	HERC5	3.0	6.33333	0.0
10	PPARD	3.0	6.5	1.5
11	ACOX1	2.0	5.83333	0.0

### 3.5. Functional enrichment analysis

To analyze the signaling pathways, the R package Bioconductor ClusterProfiler was utilized for KEGG pathway enrichment and GO analyses. The GO analysis revealed that, in terms of biological process, the overlapping genes were significantly enriched in responses to viruses, cellular responses to type I interferon, defense responses to viruses, type I interferon signaling pathways, and general responses to type I interferon (Fig. 4C). For cellular component, the genes were associated with secretory granule lumen, vacuolar lumen, cytoplasmic vesicle lumen, lysosomal lumen, tertiary granule, and late endosome (Fig. 4D). Regarding molecular function, the genes were enriched in double-stranded RNA binding, cytokine activity, MHC protein binding, cytokine receptor binding, chemokine activity, chemokine receptor binding, and CXCR chemokine receptor binding (Fig. 4E). KEGG pathway analysis indicated significant enrichment in pathways such as Influenza A, NOD-like receptor signaling, RIG-I-like receptor signaling, measles, coronavirus disease (COVID-19), Epstein–Barr virus infection, and viral protein interaction with cytokines and cytokine receptors (Fig. 4F). Among these, the NOD-like receptor signaling pathway demonstrated the highest target value, the most significant enrichment, and the lowest *P*-value.

### 3.6. Molecular docking

**Figure 5.**
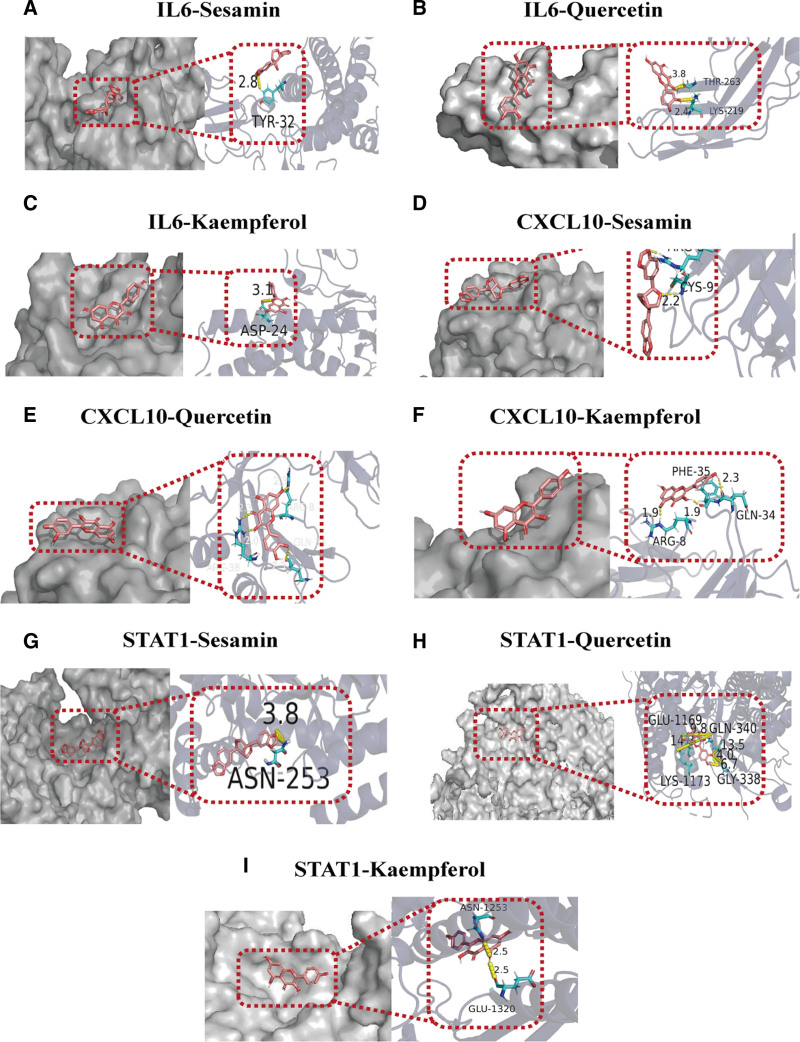
(A–I) Molecular docking maps of the Hub targets with active compounds, and the yellow dotted line is the hydrogen bond.

Molecular docking was performed using AutoDock 4.2.6 with the 3 hub genes, STAT1 (PDB:1YVL), IL-6 (PDB:1L1R), and CXCL10 (PDB:1O7Y), in combination with the 3 most active XYDN compounds. The stability of the ligand–receptor interaction increases as the binding energy decreases.^[26]^ Binding affinities below 0 kcal/mol were observed for IL-6 with Sesamin (PubChem CID:72307), Kaempferol (PubChem CID:5280863), and Quercetin (PubChem CID:5280343); CXCL10 with Sesamin, Quercetin, and Kaempferol; and STAT1 with Sesamin, Quercetin, and Kaempferol. Among these, IL-6-Quercetin, CXCL10-Kaempferol, and STAT1-Quercetin displayed the most favorable binding affinities. The molecular docking results were visualized using PyMOL 2.5 software, and the findings are illustrated in Figure 5.

### 3.7. ADMET properties

The ADMET properties of phytochemical ligands were assessed for drug-likeness, with the results summarized in Table 3. In terms of absorption, the ligands forsythiaside, ruberythric acid, and isocinchophyllamine exhibited positive CaCO_2_ permeability values. Theasinensin F and isocinchophyllamine demonstrated intestinal absorption rates exceeding 40%, which meets the desirable absorption threshold. All phytochemicals displayed skin permeability (log Kp) values below −2.5, indicating poor permeability through the skin. Based on the P-glycoprotein substrate assay, the phytochemicals were deemed compatible, suggesting their potential ability to traverse membranes via ATP-binding cassette transporters. Notably, isocinchophyllamine was identified as an inhibitor of both P-glycoprotein I and II transporters/efflux pumps. In distribution properties, forsythiaside exhibited a high steady-state volume of distribution, indicating its capacity to diffuse rapidly within the bloodstream. None of the evaluated phytochemicals showed a blood–brain barrier permeability (log BB) value exceeding 0.3, suggesting an inability to cross the blood–brain barrier. Seven cytochrome models were employed to evaluate the metabolic processing of the phytochemicals. Isocinchophyllamine was predicted to act as a substrate for CYP2D6 and CYP3A4 and as an inhibitor of CYP1A2, CYP2C19, CYP2C9, and CYP2D6. None of the tested phytochemicals were predicted to serve as substrates for the organic cation transporter 2 (OCT2). Regarding toxicity, ruberythric acid tested positive in the AMES assay, indicating a potential carcinogenic or mutagenic risk. While skin sensitization was not observed for any of the evaluated phytochemicals, isocinchophyllamine was found to be hepatotoxic.

**Table 3:** ADMET scores for the top ligands.

Property model name (unit)	Predicted value		
	Kaempferol	Quercetin	Sesamin
Absorption			
Water solubility (log mol/L)	-3.04	-2.925	-4.223
CaCO_2_ permeability (log Papp in 10 cm/s)	0.032	-0.229	1.399
Intestinal absorption (human) (% Absorbed)	74.29	77.207	97.81
Skin permeability (log Kp)	-2.735	-2.735	-2.772
P-glycoprotein substrate (yes/no)	Yes	Yes	No
P-glycoprotein I inhibitor (yes/no)	No	No	Yes
P-glycoprotein II inhibitor (yes/no)	No	No	No
Distribution			
VDss (human) (log L/kg)	1.274	1.559	-0.17
Fraction unbound (human) (Fu)	0.178	0.206	0
BBB permeability (log BB)	-0.939	-1.098	-0.862
CNS permeability (log PS)	-2.228	-3.065	-2.939
Metabolism			
CYP2D6 substrate (yes/no)	No	No	No
CYP3A4 substrate (yes/no)	No	No	Yes
CYP1A2 inhibitor (yes/no)	Yes	Yes	Yes
CYP2C19 inhibitor (yes/no)	No	No	Yes
CYP2C9 inhibitor (yes/no)	No	No	Yes
CYP2D6 inhibitor (yes/no)	No	No	No
CYP3A4 inhibitor (yes/no)	No	No	Yes
Excretion			
Total clearance (log mL/min/kg)	0.477	0.407	-0.126
Renal OCT2 substrate (yes/no)	No	No	No
Toxicity			
AMES toxicity (yes/no)	No	No	Yes
Max. tolerated dose (human) (log mg/kg/day)	0.531	0.499	0.089
hERG I inhibitor (yes/no)	No	No	No
hERG II inhibitor (yes/no)	No	No	No
Oral rat acute toxicity (LD50) (mol/kg)	2.449	2.471	2.883
Oral rat chronic toxicity (LOAEL) (log mg/kg_bw/day)	2.505	2.612	1.568
Hepatotoxicity (yes/no)	No	No	No
Skin sensitization (yes/no)	No	No	No
T. Pyriformis toxicity (log µg/L)	0.312	0.288	0.34
Minnow toxicity (log mM)	2.885	3.721	0.039

ADMET = absorption, distribution, metabolism, excretion, and toxicity, BBB = blood–brain barrier, OCT2 = organic cation transporter 2, VDss = steady-state volume of distribution,

### 3.8. Molecular dynamics simulation

The investigation focused primarily on the IL-6–Quercetin, CXCL10–Kaempferol, and STAT1–Quercetin complexes based on the degree values obtained from molecular docking and network analysis. To evaluate the stability of ligand molecules and their interactions with STAT1, IL-6, and CXCL10 proteins, all-atom MD simulations lasting 50 ns were performed using GROMACS. Key metrics such as solvent-accessible surface area (SASA), RMSD, RMSF, hydrogen bonds, and the radius of gyration (Rg) were extracted from the MD simulations to assess the stability of the docked complexes.

**Figure 6.**
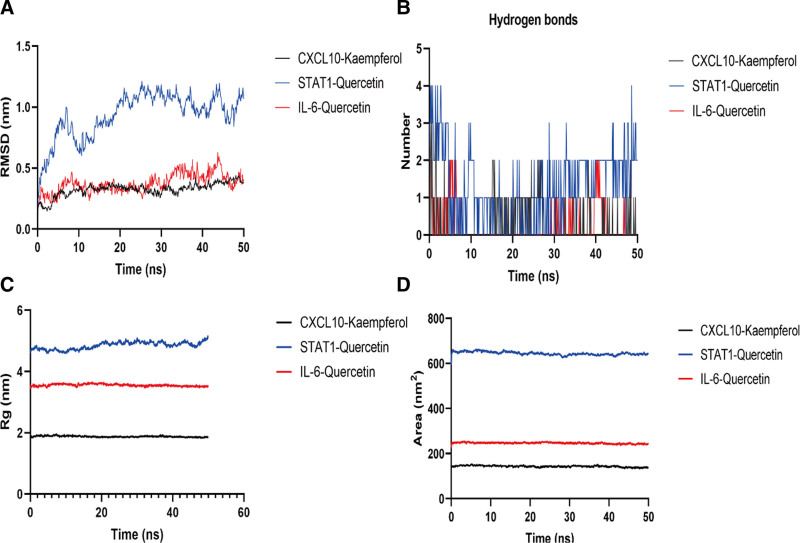
(A) RMSD of IL-6–Quercetin, CXCL10–Kaempferol and STAT1–Quercetin. (B) Hydrogen bonds of IL-6–Quercetin, CXCL10–Kaempferol, and STAT1–Quercetin. (C) Rg of backbone atoms. (D) SASA of IL-6–Quercetin, CXCL10–Kaempferol, and STAT1–Quercetin. IL-6 = interleukin-6, SASA = solvent-accessible surface area.

Protein–ligand RMSD is a widely used measure for comparing the structural similarity of 2 protein–ligand complexes. It calculates the positional differences between corresponding atoms in the complexes after aligning them to a common reference frame.^[27]^ RMSD is computed as the square root of the average squared positional differences of ligand atoms, while keeping the protein atoms fixed. A low RMSD value indicates structural similarity, while a high value reflects dissimilarity. As depicted in Figure 6A, the RMSD during the 20 ns simulation indicated protein stability, confirming that the MD simulation trajectories could be utilized for further analysis. Between the 20 to 50 ns time frame, the RMSD of the IL-6–Quercetin and CXCL10–Kaempferol complexes was lower than that of the STAT1–Quercetin complex, suggesting greater structural stability for these interactions.

Hydrogen bonds were calculated for each system throughout the 100 ns simulation to provide a detailed characterization of the interactions between ligand and protein atoms during the MD trajectories. The results demonstrated that hydrogen bonds within the complexes were consistently maintained throughout the simulation process (Fig. 6B), highlighting their stability during the dynamics modeling.

When describing the general size and shape of a protein–ligand complex, the Rg is an important measure. It is worth noting that the ligand typically contributes minimally to the overall Rg due to its smaller size relative to the protein. The Rg of a protein–ligand complex is determined by the combined shape, size, and specific interactions between the ligand and the protein. This metric serves as a useful indicator for guiding drug design and assessing the binding affinity and stability of the complex. As shown in Fig. 6C, all complexes displayed Rg values within an acceptable range, indicating strong stability. Each ligand exhibited consistent values for the radius of gyration.

To evaluate binding interactions within a protein–ligand complex, SASA measures the molecule’s surface area that is accessible to solvent molecules. SASA can also provide insights into changes in molecular exposure or conformation over the course of a simulation. The SASA values of the tested compounds showed minimal fluctuations during the simulation, reflecting stable interactions with their respective protein complexes (Fig. 6D).

**Figure 7.**
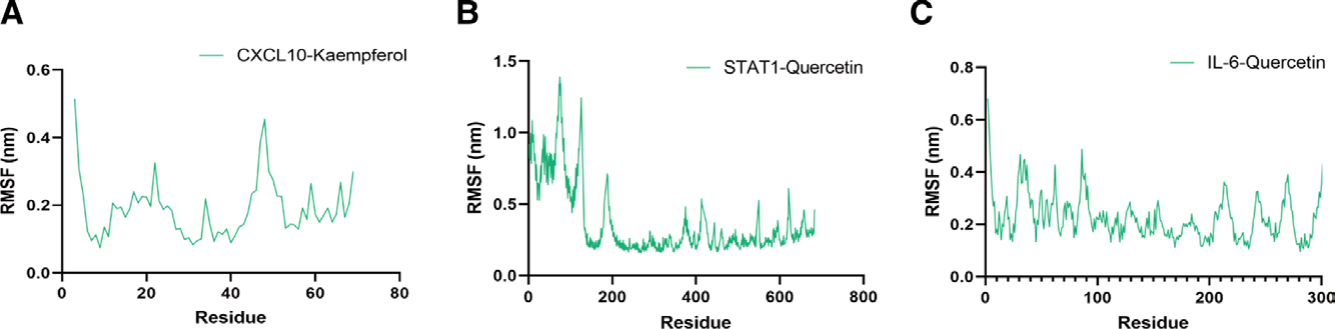
(A–C) RMSF of backbone atoms.

The RMSF is a measure of the structural mobility or flexibility of a protein. It calculates the average deviation of each protein atom from its mean position during a specified simulation period. RMSF provides valuable insights into protein dynamics, such as conformational changes or protein–ligand interactions. The RMSF values are plotted as a function of the residue number, highlighting regions of high flexibility or rigidity within the protein structure. Regions with low RMSF values correspond to stable or rigid areas, while high RMSF values typically indicate flexible regions, such as exposed surface areas or loop regions. In the STAT1–Quercetin model, the RMSF analysis revealed significant fluctuations in the amino acid residues located in loop regions, indicating high flexibility in these areas. These fluctuations suggest notable structural variability in these regions during the simulation, primarily due to disruptions in amino acid strands. In contrast, the CXCL10–Kaempferol and IL-6–Quercetin complexes exhibited only minor conformational changes, which supports their structural stability (Fig. 7).

Overall, the findings from the MD simulations were consistent with molecular docking results, reinforcing the efficient binding interactions of CXCL10–Kaempferol and IL-6–Quercetin.

## 4. Discussion

The hepatotropic, non-cytopathic HCV is highly proficient at evading the host immune system.^[28]^ Following infection, innate immunity pathways in hepatocytes are activated, leading to the infiltration of pro-inflammatory and antiviral immune effector cells into the liver.^[29]^ According to the WHO, 3 to 4 million new HCV infections occur annually worldwide. Among those with chronic infections, an estimated 130 to 170 million individuals face an elevated risk of developing hepatocellular carcinoma, liver cirrhosis, or both. These complications result in over 350,000 deaths annually.^[30]^ The prevalence of HCV varies significantly by region, with northern Europe showing infection rates as low as 0.1% to 1%, while southern Europe experiences rates of 2.5% to 3.5%. Egypt has the highest prevalence, with 22% of its population affected. Pakistan and China follow, each with infection rates exceeding 3%.^[31]^

Since 1991, interferon (IFN) monotherapy has been used to treat HCV, evolving into IFN combined with ribavirin and later into pegylated IFN with ribavirin, which became the standard treatment.^[32–34]^ However, a subset of patients fails to achieve a satisfactory response to antiviral therapy. Although newer oral antiviral medications promise better outcomes, they remain difficult to access, extremely expensive, and sometimes associated with complications, discouraging many patients from pursuing treatment. Additionally, other antiviral therapies often face issues such as limited efficacy, relapse after treatment discontinuation, and significant adverse effects, further complicating the management of HCV.

TCM has been a unique and valuable health resource for over 2000 years.^[35]^ Known for its broad range of targets, significant efficacy, and favorable safety profile, TCM holds promise for alleviating symptoms and preventing the progression of diseases like hepatitis C.^[36]^ Over time, TCM researchers have diligently investigated the etiology and pathogenesis of hepatitis C, developing various treatment approaches and prescriptions. Improving diagnostic and therapeutic strategies for hepatitis C has been a major focus of research both domestically and internationally. However, effective treatments with robust evidence of efficacy remain limited, highlighting the urgent need for therapeutic options that provide liver protection with fewer side effects to meet clinical demands.

XYDN, originally developed as an in-hospital preparation by the Uyghur Hospital of Xinjiang Uyghur Autonomous Region, is now exclusively manufactured by the Xinjiang Uyghur Autonomous Region Uyghur Pharmaceutical Industry Limited Liability Company. This natural medicine is specifically formulated to address gallbladder and liver disorders, including acute and chronic hepatitis, jaundice hepatitis, acute and chronic cholecystitis, and acute and chronic cholangitis.^[37,38]^ Clinical studies have demonstrated that XYDN possesses laxative and detoxifying properties, promotes liver cleansing and soothing, reduces inflammation, and alleviates pain. With a history spanning hundreds of years, its efficacy has proven to be both consistent and significant.^[39]^

Experimental research conducted by Ai et al demonstrated that oral administration of XYDN significantly lowered serum GPT and GOT levels in mice with acute liver injury induced by CCL4 and D-GAL.^[40]^ The treatment also increased hepatic CSH-Px activity and bile flow. Immunological studies further revealed that XYDN syrup exhibits significant immunosuppressive effects. Between 1996 and 2001, the Uyghur Hospital of Xinjiang treated 100 cases of liver and gallbladder disorders using XYDN syrup. Results indicated a 60% reduction in ALT and AST levels in 48.4% of patients with elevated liver enzymes.^[41,42]^ Among hyperlipidemic patients, 42.4% showed a reduction in cholesterol levels exceeding 24%, while 29.7% experienced a decrease in triglyceride levels exceeding 21%, suggesting that XYDN also exerts lipid-lowering effects. Research by Wang et al further emphasized that XYDN effectively treats cholestasis-related jaundice and increases SOD enzyme activity in the liver, thereby boosting hepatocyte antioxidative capacity, scavenging free radicals, and preventing liver fibrosis. Additional experimental findings indicated that XYDN mitigates weight loss and liver enlargement associated with liver damage, significantly reduces serum ALT and AST levels, and restores liver tissue MDA, SOD, and GSH-PX levels. Its efficacy in reducing ALT and AST levels has been particularly notable.^[43–45]^

As a complex TCM formulation, XYDN presents challenges for conventional pharmacological research, making its mechanisms of action difficult to fully understand. Deciphering the molecular mechanisms and intrinsic synergism of XYDN in treating hepatitis C is vital for providing a scientific foundation to TCM theories and expanding its clinical applications. Network pharmacology offers a powerful approach by constructing network models that translate intricate biological interactions into visual representations. This method facilitates the identification and understanding of complex biological systems, offering valuable insights into the mechanisms underlying TCM compounds.

The compound–target network offers a visual framework for understanding the interactions between bioactive compounds and their molecular targets, which are closely associated with the pathogenesis of specific diseases. This approach enables a comprehensive and integrated view of the pharmacological mechanisms at play. In this study, 22 active compounds from XYDN were identified, corresponding to 260 molecular targets. Among these, Quercetin, Sesamin, and Kaempferol emerged as key compounds with higher degree values based on network data analysis. Phenolic and flavonoid compounds, including sesamin, astragalin, gallic acid, vanillic acid, and apigenin, demonstrated notable antioxidant properties and regulatory effects on viral replication pathways, making them promising candidates for hepatoprotection. Phenolic compounds such as Kaempferol, ellagic acid, myricitrin, and Quercetin have been shown to exhibit antiviral activity against a wide range of viruses.^[46]^ Their antiviral effects are primarily mediated through interactions with viral and host proteins and enzymes, which disrupt viral replication and infectivity. Studies by researchers including Loaiza-Cano and Xu have extensively documented the antiviral properties of these compounds.^[47,48]^ Screening over 100 phenolic compounds revealed that those with abundant hydroxyl (-OH) groups and specific structural arrangements exhibited the strongest inhibitory effects.

Although these hepatoprotective compounds may not fully eradicate the underlying viral infection, their targeted application at higher dietary concentrations could help counteract, delay, or reduce symptoms associated with HBV in unvaccinated individuals. This could occur through their roles as synergists, agonists, or antagonists, achieved by regulating protein and enzymatic pathways.^[49,50]^ Quercetin, for instance, functions as an antagonist, inhibiting HCV replication by targeting the multi-enzymatic activities of NS5B, envelope glycoprotein 2, and nonstructural protein 3 (NS3) protease. In silico molecular docking studies have further revealed that flavonoids such as apigenin, Quercetin, and luteolin exhibit comparable binding energies to synthetic NS5B inhibitors like sofosbuvir.^[51]^

GEO data analysis identified 394 DEGs associated with HCV, and target screening revealed 12 overlapping genes linked to medications, illnesses, and DEGs as key therapeutic targets. PPI networks play a crucial role in revealing biological functions and mechanisms. Similar to other biological networks, PPI networks can be analyzed using topological parameters such as degree, betweenness, and closeness centrality. These metrics help prioritize nodes, identify central hubs that regulate disease-associated pathways, and shed light on interactions and their functional implications under pathological conditions. In this study, CXCL10, IL-6, and STAT1 were identified as central targets within the PPI network. To investigate the role of these targets in HCV pathogenesis and align them with the antiviral properties of medicinal plants, pathway enrichment analysis was performed based on the common predicted targets. KEGG pathway analysis highlighted several pathways associated with HCV and related genes, including those encompassing key predicted targets and other relevant pathways.

Our findings, consistent with earlier studies, highlight the critical role of chemokines in the pathophysiology of HCV infection. Specific chemokines, such as chemokine (CXC motif) ligand 2 (CCL2), CXC chemokine ligand 10 (CXCL10), and CX3C chemokine ligand 1 (CX3CL1), have been implicated in the progression of this disease. CXCL10, also known as interferon-inducible protein-10 (IP10), acts as a potent chemoattractant, recruiting lymphocytes to HCV-infected tissues.^[52]^ Among the elevated chemokines observed in HCV-positive patients, CXCL10 stands out as a potential diagnostic biomarker. Recent studies have shown a strong correlation between CXCL10 expression and the degree of apoptosis in hepatitis C patients, a relationship mediated through TLR-4 activation, a noncognate receptor.^[53]^ Additionally, CXCL10 levels in intrahepatic and serum samples have been consistently linked to the severity of HCV-induced liver fibrosis.^[54]^ In HCV-infected individuals, circulating CXCL10 also serves as an independent biomarker for predicting the recurrence of severe fibrosis after liver transplantation.^[55]^

IL-6, a key cytokine during inflammation, plays a critical role in the body’s immune response. In the liver, it is the primary cytokine responsible for significantly altering the synthesis of many APP.^[56]^ As a multifunctional cytokine, IL-6 influences a variety of cellular activities, including proliferation, survival, apoptosis, and the regulation of inflammatory responses.^[57]^ During hepatitis C infection, IL-6 production increases significantly in B cells alongside interferon-beta, following strong activation of toll-like receptors.

To protect hepatocytes from viral infections, the induction of IFNα is crucial. This process involves the activation of interferon-stimulated genes (ISGs), which regulate viral replication via the Janus kinase–signal transducer (JAK–STAT) pathway. HCV and alcohol use various mechanisms to disrupt the IFN-induced JAK–STAT1 signaling pathway, including inhibiting STAT1 methylation^[58]^ and suppressing STAT1 phosphorylation.^[59,60]^ These disruptions are linked to changes in STAT1 arginine methylation, as HCV has been shown to inhibit the methylation of STAT1 at Arg-31 in infected hepatocytes.^[61,62]^ In a study by Wenyu Lin et al, IFNα was found to promote the activation of ISGs, with OASL and OAS-1 used as representative genes. Silencing JMJD6 reduced HCV RNA levels by suppressing ISG activation, whereas overexpression of JMJD6 in HCV-infected Huh7.5 cells decreased ISG activation by IFNα and increased HCV RNA levels. These findings suggest that JMJD6 regulates HCV infectivity in hepatocytes by regulating the JAK–STAT1 pathway and suppressing IFNα-dependent ISG activation.^[63]^

Molecular docking is a widely used method in drug discovery to predict interactions between molecules and biological targets. In this study, the interactions of Quercetin, Sesamin, and Kaempferol with 3 core targets associated with HCV pathogenesis were analyzed. Quercetin showed the highest binding affinity for IL-6 and STAT1, while kaempferol exhibited the strongest binding affinity for CXCL10. However, it is important to note that molecular docking primarily estimates the compatibility of compounds at a protein’s active site.

The reliance on binding conformation data has increased with the use of MD simulations and derived binding energy values to evaluate compound–protein target systems. MD simulations provide valuable insights into the dynamics of docked complexes, enabling the analysis of energy landscape fluctuations and offering a better understanding of complex stability and ligand-induced protein conformational changes.^[64]^ In summary, the combination of MD simulations and molecular docking in this study revealed significant binding affinities between protein matrices and active compounds.

In conclusion, this investigation establishes a strong scientific foundation for understanding the multi-target mechanisms of XYDN as a potential treatment for HCV. By integrating bioinformatics techniques with network pharmacology, key biological pathways and interactions underlying HCV pathogenesis were identified, along with putative therapeutic targets that could be regulated to treat the disease. Although the study’s conclusions were validated through MD simulations and molecular docking, further in vitro and in vivo studies are required to confirm these findings.

This research has several limitations. First, additional experimental validation is needed to strengthen the current findings. Second, improving the accuracy of network pharmacology analysis requires access to a more comprehensive and robust database of conventional drugs and target genes. Third, while the integration of network pharmacology and molecular docking offers valuable insights, the precise therapeutic mechanism of XYDN in treating HCV remains only partially understood. To address these gaps, a multidisciplinary approach is essential for fully uncovering the mechanisms through which XYDN acts against HCV.

## 5. Conclusion

The development of effective strategies for preventing, detecting, and treating HCV infection and its associated complications is critical, given the high global prevalence of HCV. This urgency highlights the need for innovative and effective therapies for HCV infection. In this context, our research introduces a novel scientific approach for evaluating the multi-component and multi-target effects of XYDN’s active compounds. This study identified Quercetin, Kaempferol, and Sesamin as promising candidates for HCV treatment by combining bioinformatics and network pharmacology techniques. Additionally, the findings highlighted IL-6, CXCL10, and STAT1 as viable therapeutic targets to reduce cell proliferation and growth associated with HCV. In summary, this research contributes to a deeper understanding of the chemical composition of XYDN and how its active compounds work synergistically to combat HCV.

## Acknowledgments

We are grateful to all the researchers who provided the data we used.

## Author contributions

**Conceptualization:** Dilihuma Dilimulati, Nuerbiye Nueraihemaiti.

**Data curation:** Sendaer Hailati, Mengyuan Han.

**Formal analysis:** Alhar Baishan.

**Funding acquisition:** Wenting Zhou.

**Investigation:** Dilihuma Dilimulati, Nuerbiye Nueraihemaiti, Alfira Aikebaier.

**Methodology:** Dilihuma Dilimulati.

**Project administration:** Wenting Zhou.

**Resources:** Sendaer Hailati.

**Software:** Kayisaier Abudurousuli.

**Supervision:** Maidina Abulizi.

**Validation:** Dilihuma Dilimulati.

**Visualization:** Nuerbiye Nueraihemaiti, Nulibiya Maihemuti.

**Writing – original draft:** Dilihuma Dilimulati, Nuerbiye Nueraihemaiti.

**Writing – review & editing:** Wenting Zhou.
